# An artificial intelligence based app for skin cancer detection evaluated in a population based setting

**DOI:** 10.1038/s41746-023-00831-w

**Published:** 2023-05-20

**Authors:** Anna M. Smak Gregoor, Tobias E. Sangers, Lytske J. Bakker, Loes Hollestein, Carin A. Uyl – de Groot, Tamar Nijsten, Marlies Wakkee

**Affiliations:** 1 Department of Dermatology, Erasmus MC Cancer Institute, University Medical Center Rotterdam, Rotterdam, The Netherlands; 2 Erasmus School of Health Policy & Management, Erasmus University Rotterdam, Rotterdam, The Netherlands

**Keywords:** Melanoma, Basal cell carcinoma, Squamous cell carcinoma, Health care economics, Cancer screening

## Abstract

Artificial intelligence (AI) based algorithms for classification of suspicious skin lesions have been implemented in mobile phone apps (mHealth), but their effect on healthcare systems is undocumented. In 2019, a large Dutch health insurance company offered 2.2 million adults free access to an mHealth app for skin cancer detection. To study the impact on dermatological healthcare consumption, we conducted a retrospective population-based pragmatic study. We matched 18,960 mHealth-users who completed at least one successful assessment with the app to 56,880 controls who did not use the app and calculated odds ratios (OR) to compare dermatological claims between both groups in the first year after granting free access. A short-term cost-effectiveness analysis was performed to determine the cost per additional detected (pre)malignancy. Here we report that mHealth-users had more claims for (pre)malignant skin lesions than controls (6.0% vs 4.6%, OR 1.3 (95% CI 1.2–1.4)) and also a more than threefold higher risk of claims for benign skin tumors and nevi (5.9% vs 1.7%, OR 3.7 (95% CI 3.4–4.1)). The costs of detecting one additional (pre)malignant skin lesion with the app compared to the current standard of care were €2567. Based on these results, AI in mHealth appears to have a positive impact on detecting more cutaneous (pre)malignancies, but this should be balanced against the for now stronger increase in care consumption for benign skin tumors and nevi.

## Introduction

Skin cancer is one of the most common types of cancer and the incidence is rising, posing a major burden for healthcare systems^1–4^. Technological advances in medicine, such as teledermatology and mobile phone (mHealth) apps using artificial intelligence (AI) are being implemented in clinical care as a possible solution to reduce this burden. By lowering the amount of consultations for benign skin lesions and increasing the likelihood of early detection of skin cancer, implementation of AI could reduce pressure on clinicians and additionally reduce related healthcare costs^5^.

Even though the concept of AI-based algorithms for skin cancer detection shows a lot of potential, this has only been investigated in a sterile research setting. And while AI can perform on par to dermatologists in recognizing skin cancer on dermoscopy-based pictures^6–8^, it is still uncertain how and for whom it should be implemented in clinical care. In recent years, these AI-based algorithms for skin cancer detection have been implemented in several mHealth apps making this technique accessible to the general population^9^. The Netherlands is facing a unique position, where integration of AI-based mHealth apps in a population-based setting has rapidly progressed. Several large health insurance companies are reimbursing an mHealth app for skin cancer detection for their insurees^10^, enabling laypersons to use an AI-based mHealth app to evaluate whether they should visit a general practitioner (GP) for a potentially cancerous skin lesion. These real world data can help to better understand the potential impact on healthcare consumption by assessing how it affects clinicians and patients when they actually use such an app^11^. Therefore, this study aims to evaluate the impact of an mHealth app for suspicious skin lesions on dermatological healthcare consumption in a population based setting.

## Results

### Demographics and app usage

**Fig. 1:**
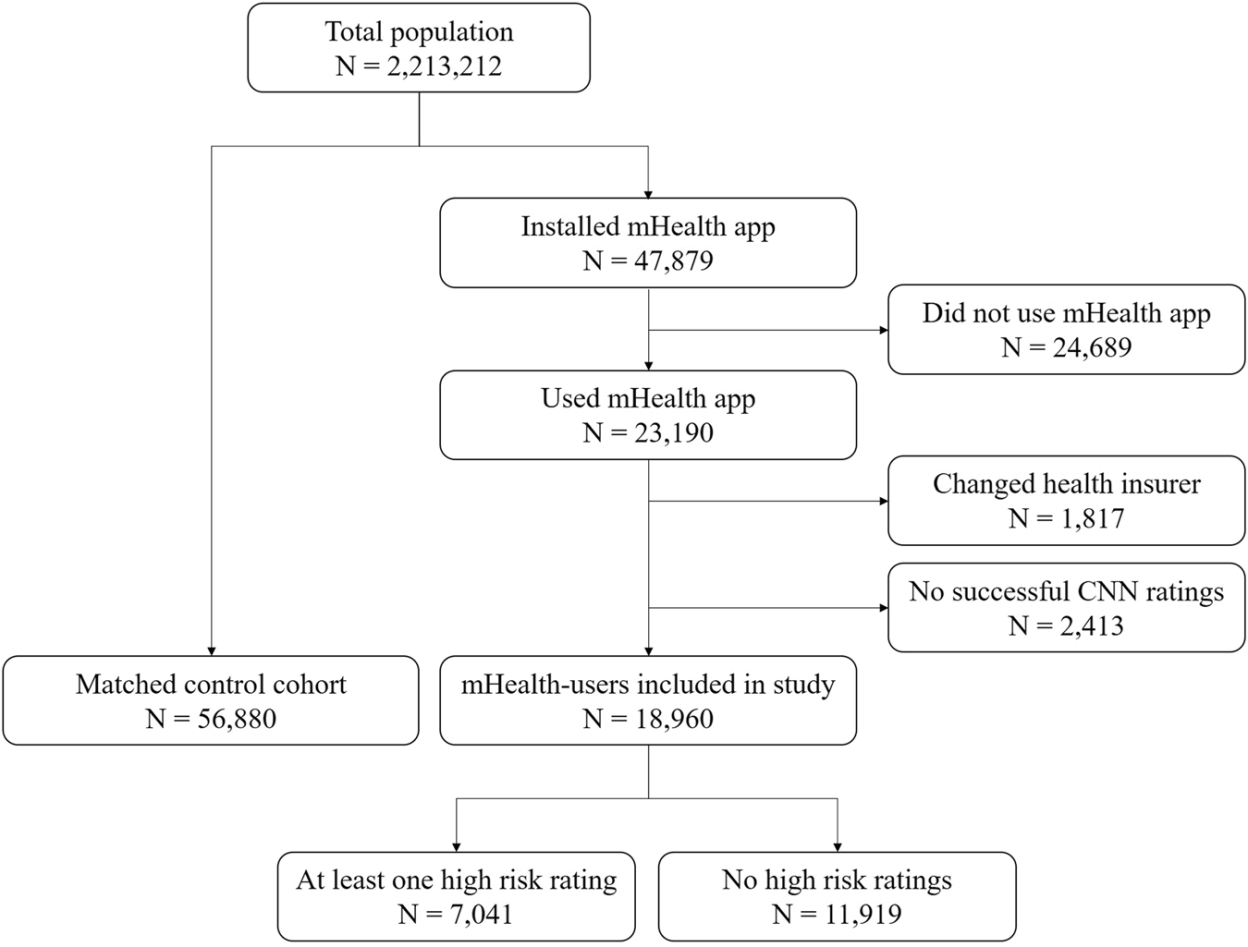
Flowchart of the study design. mHealth mobile health, CNN Convolutional neural network.

Of the 2,213,212 adults invited to use the mHealth app, 47,879 individuals (2.0%) installed the app and 20,777 (0.9%) had at least one successful assessment of a skin lesion. Of these 20,777 insurees, 12 months of claims data were available of 18,960 participants (Fig. 1). The mHealth-users were matched to 56,880 controls of whom 88.3% (*n* = 50,197) could be matched on all matching criteria. Users of the app and their controls had a mean age of 48.4 years (SD 14.0), 53.2% were female, and 42.6% were classified as middle SES (Table 1). The majority did not have a history of skin cancer (95.3%) and were healthy individuals (median number of comorbidities 0 (IQR 0-0)).

**Table 1:** Baseline characteristics of mHealth-users.

	mHealth-users (*n* = 18,960)
Age (years, SD)	48.4 (14.0)
Sex, *n* (%)	
Male	8882 (46.8)
Female	10,078 (53.2)
SES-status, *n* (%)^a^	
Low	4368 (23.0)
Middle	8086 (42.6)
High	6288 (33.2)
Missing	218 (1.1)
Skin (Pre-)Malignancy in medical history, *n* (%)	885 (4.7)
No. of Co-morbidities, median (IQR)	0 (0-0)
Algorithm assessments, *n* (%)	64,128
Low risk	37,500(58.5)
Low risk with symptoms	8490 (13.2)
High risk	15,083 (23.5)
Failed	3055 (4.8)

Values are mean with standard deviation, median with range or number of cases with percentages. Co-morbidities are based on medications on the ATC 5^th^ level.

*SD* standard deviation, *SES* social economic status, *no.* number, *IQR* interquartile range.

^a^Percentages do not add up to 100% due to rounding differences.

The 18,960 users performed a total of 64,128 assessments. Of those, the CNN rated 45,990 (71.7%) as low risk, 15,083 (23.5%) as high risk, and 3055 (4.8%) could not be evaluated. A panel of teledermatologists upgraded 1277 low risk images (2.8%) to high risk, and 5098 high risk images (33.8%) were downgraded to low risk.

### Healthcare consumption related to skin lesions

The proportion of claims for premalignant and malignant skin lesions combined was significantly higher for mHealth-users (6.0%) than for controls (4.6%, OR 1.3 (95% CI 1.2–1.4)) (Table 2). When evaluating claims for premalignancies and malignancies separately, the majority were related to malignant lesions in the group of mHealth-users (773 out of 1164) and controls (1785 out of 2681), resulting in a similar OR of 1.3 (95% CI 1.2–1.4). There was an even stronger difference in the proportion of claims for benign skin tumors and nevi between mHealth-users (5.9%) and controls (1.7%), resulting in an almost fourfold higher likelihood of mHealth-users having these claims (OR 3.7 (95% CI 3.4–4.1)). The majority of this effect was due to claims for nevi for mHealth-user (850 out of 1117) and controls (667 out of 946). When evaluated separately, this resulted in an even larger likelihood of mHealth-users (4.5%) to get a claim for nevi compared to controls (1.2%)(OR 4.0 (95% CI 3.6–4.4)). Dermatological claims unrelated to usage of the app were similar between both groups (OR 1.1 (95% CI 1.0–1.2)).

**Table 2:** Proportions of dermatological healthcare claims for users of the mHealth app (*n* = 18,960) and a matched cohort (*n* = 56,880).

	Matched controls (*n* = 56,880)	mHealth-users (*n* = 18,960)	*p*-value
Premalignant and malignant skin lesions			
Premalignant skin lesions			
Percentage, % (*n*)	1.58 (896)	2.06 (391)	<0.001
Odds Ratio (OR) (95% CI)	Ref	1.32 (1.16–1.49)	<0.001
Malignant skin lesions			
Percentage, % (*n*)	3.14 (1785)	4.08 (773)	<0.001
Odds Ratio (OR) (95% CI)	Ref	1.31 (1.20–1.43)	<0.001
Nevi and Benign skin tumors			
Nevi			
Percentage, % (*n*)	1.17 (667)	4.48 (850)	<0.001
Odds Ratio (OR) (95% CI)	Ref	3.96 (3.57–4.39)	<0.001
Benign skin tumors			
Percentage, % (*n*)	0.49 (279)	1.41 (267)	<0.001
Odds Ratio (OR) (95% CI)	Ref	2.90 (2.44–3.44)	<0.001
Unrelated dermatological claims			
Percentage, % (*n*)	4.92 (2800)	5.28 (1001)	=0.066
Odds Ratio (OR) (95% CI)	Ref	1.08 (1.00–1.16)	=0.066

Percentages are number of people with a claim per subcategory of claims. *P*-values are the difference in proportion of claims, calculated using a two proportions z-test or corresponding odds ratio’s using Fisher’s Exact Test for Count Data.

*CI* confidence interval, *OR* Odds Ratio, *Ref* Reference.

During the study period, mHealth-users had twice as many biopsies and excisions at the GP than the matched controls, respectively 75 claims per 1000 persons versus 34 claims per 1000 persons (*p* < 0.001) (Table 3). A similar pattern emerged when comparing hospital-based excisions (55 vs. 25 claims per 1000 persons, *p* < 0.001).

**Table 3:** Differences in proportion of dermatology related diagnostic and therapeutic interventions in 2019 for users of the mHealth app (*n* = 18,960) and a matched cohort (*n* = 56,880).

	Matched controls (*n* = 15,404 claims)	mHealth-users (*n* = 8287 claims)	
Primary care			
Biopsy or Excision at GP			
Percentage, % (*n*)	12.99 (1913)	17.91 (1424)	*p* < 0.001
No of claims per 1000 persons	33.63	75.11	Δ 41.47
Teledermatology at GP			
Percentage, % (*n*)	0.89 (131)	1.25 (99)	*p* = 0.017
No of claims per 1000 persons	2.30	5.22	Δ 2.92
Secondary care			
Mohs surgery			
Percentage, % (*n*)	0.65 (95)	0.47 (37)	*p* = 0.117
No of claims per 1000 persons	1.67	1.95	Δ 0.28
Excision in hospital setting, benign lesions			
Percentage, % (*n*)	1.87 (276)	4.67 (371)	*p* < 0.001
No of claims per 1000 persons	4.85	19.57	Δ 14.72
Excision in hospital setting, (pre)malign lesions			
Percentage, % (*n*)	7.67 (1129)	8.31 (661)	*p* = 0.108
No of claims per 1000 persons	19.85	34.86	Δ 15.01

Biopsies or excisions performed in primary care are done by the general practitioner. The described excisions in a hospital setting were done by a dermatologist. Values are presented as a percentage of claims per group compared to the absolute amount of claims in the year, or absolute number of claims per 1000 persons. *P*-values are the difference in proportion of claims calculated using a two proportions z-test. Delta is the absolute difference in number of claims per 1000 persons.

*GP* General Practitioner, *No.* Number.

### Subgroup analyses

Among the 18,960 mHealth-users, 7041 (37.1%) had at least one high risk assessment and 11,919 (62.9%) had only low risk assessments (Table 4). Of all mHealth-users with at least one high risk assessment, 9.1% eventually had a claim for a (pre)malignancy, while this was 4.3% among those with only low risk assessments. The odds of having a claim for a (pre)malignant skin lesion was 1.6 times higher among those with a high risk assessment compared to their controls (OR 1.6 (95% CI 1.4–1.8)). This high risk group also had a six fold higher risk of having a claim for benign skin tumors and/or nevi (OR 6.7 (95% CI 5.9–7.7)). Curiously, the likelihood of having a claim for a benign skin tumor or nevus was also higher for mHealth-users who only received low risk assessments compared to their matched controls (OR 2.1 (95% CI 1.9–2.4)).

**Table 4:** Proportions of dermatological healthcare claims for users of the mHealth app (*n* = 18,960) and a matched cohort (*n* = 56,880) stratified per mHealth-users with at least one high risk assessment and users with only low risk assessments.

High risk			
	Matched controls (*n* = 21,123)	High risk (*n* = 7041)	*p*-value
Premalignant skin lesions			
Percentage, % (*n*)	1.89 (399)	2.95 (208)	<0.001
Odds Ratio (OR) (95% CI)	Ref	1.58 (1.33–1.88)	<0.001
Malignant skin lesions			
Percentage, % (*n*)	4.06 (858)	6.28 (442)	<0.001
Odds Ratio (OR) (95% CI)	Ref	1.58 (1.40–1.78)	<0.001
Nevi and Benign skin tumors			
Percentage, % (*n*)	1.60 (339)	9.87 (695)	<0.001
Odds Ratio (OR) (95% CI)	Ref	6.71 (5.87–7.69)	<0.001
Unrelated dermatological claims			
Percentage, % (*n*)	4.88 (1031)	6.04 (425)	<0.001
Odds Ratio (OR) (95% CI)	Ref	1.25 (1.11–1.41)	<0.001
Low risk			
	Matched controls (*n* = 35,757)	Low risk (*n* = 11,919)	*p*-value
Premalignant skin lesions			
Percentage, % (*n*)	1.39 (497)	1.54 (183)	0.333
Odds Ratio (OR) (95% CI)	Ref	1.11 (0.93–1.31)	0.333
Malignant skin lesions			
Percentage, % (*n*)	2.59 (927)	2.78 (331)	0.3326
Odds Ratio (OR) (95% CI)	Ref	1.07 (0.94–1.22)	0.3326
Nevi and Benign skin tumors			
Percentage, % (*n*)	1.68 (602)	3.52 (419)	<0.001
Odds Ratio (OR) (95% CI)	Ref	2.13 (1.87–2.42)	<0.001
Unrelated dermatological claims			
Percentage, % (*n*)	4.95 (1769)	4.83 (576)	0.634
Odds Ratio (OR) (95% CI)	Ref	0.98 (0.88–1.08)	0.634

A high risk rating was defined as either the CNN or teledermatologist rated a photo as high risk. Low risk was defined as neither the CNN nor the teledermatologist rated a photo as high risk. Percentages are number of people with a claim per subcategory of claims. *P*-values are the difference in proportion of claims, calculated using a two proportions z-test or corresponding odds ratio’s using Fisher’s Exact Test for Count Data.

*CI* confidence interval, *OR* Odds Ratio, *Ref* Reference.

A small subgroup of mHealth-users (4.7%) had a positive medical history for either a malignant or premalignant skin lesion. When evaluating the difference in claims for this high risk group of mHealth-user and their matched controls, we found that mHealth-users had less claims for malignant skin lesions (OR 0.83 (95% CI 0.71–0.97)) and also less claims for dermatologic conditions unrelated to app-usage (OR 0.73 (95% CI 0.57–0.94)) (Supplementary Table 1). The number of claims for premalignant skin lesions, nevi, and benign skin tumors was similar between both groups.

### Cost-effectiveness analysis

Users of the app were responsible for a higher amount of average annual dermatological healthcare costs per person, i.e. €64.97 vs. €43.09 (Δ €21.88 (95% CI 17.90–25.85), *p* < 0.001), corresponding with the larger amount of claims (Supplementary Table 2). The largest difference in average healthcare costs was attributed to consultations for nevi (€11.05 per person vs €2.71, *p* < 0.001) and premalignant or malignant skin neoplasia (€31.01 per person vs €20.88, *p* < 0.001). Costs for unrelated dermatological claims were comparable between mHealth-users and the matched cohort (€20.01 per person vs €18.47, *p* = 0.211). Mean costs per subtype of claim were similar between both groups, with the exception of malignant skin lesions, where the mean costs per claim for a malignant skin lesion were higher for mHealth-users (€613.36 vs €520.05, *p* < 0.001) (Supplementary Table 3, Supplementary Fig. 1).

**Fig. 2:**
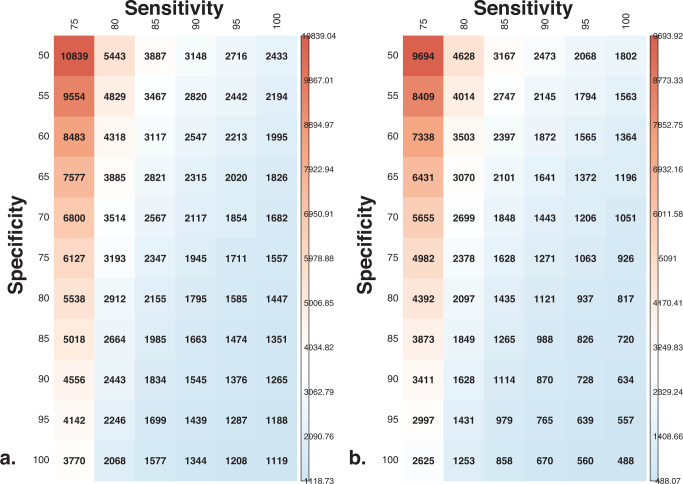
Simulation of the Incremental Cost-effectiveness Ratio (ICER) with different combinations of sensitivity and specificity and costs in euros. **a** Including costs for premalignant, malignant and benign skin lesions. **b** Including only costs for benign skin lesions.

The ICER provides a cross-sectional estimate of the costs for detecting one new skin premalignancy or malignancy, without taking into account the long-term effects. In the study cohort the ICER was €2567 per additional (pre)malignant skin lesion detected by the app compared to the current standard of care, which also includes the treatment of the additionally detected (pre)malignancies. The ICER representing the cost for additionally detected benign skin tumors and nevi per detected (pre)malignant skin lesion was €1843. For an app with a perfect detection accuracy (sensitivity and specificity 100%), the estimated ICER would have been €1119 per additionally detected (pre)malignant skin lesion when including all costs (Fig. 2a). Only accounting for the costs of benign skin tumors and nevi, the ICER would have been €488 per additionally detected (pre)malignant skin lesion at sensitivity and specificity 100% (Fig. 2b). Variation in the costs to detect one new skin premalignancy or malignancy shows a wide range, depending on the accuracy of the app, and can be as low €488 to as high as €10,839.

## Discussion

To our knowledge this is the first study evaluating the impact of an AI-based mHealth app for skin cancer detection on dermatological healthcare consumption in the general population. Among people who actually used the app, there was a 32% increase in claims for (pre)malignant skin lesions compared to a comparable group who did not use the mHealth app. This effect was however counterbalanced by a three to fourfold higher risk of claims for benign skin tumors and nevi among the mHealth-users as well.

These findings were to be expected, based on the previous reported diagnostic accuracy of the examined app^12,13^. They are also in line with other widely accepted population-based cancer screening programs that balance between correct diagnosis of malignancies and false positive results^14^ and current clinical dermatological practice, where for every melanoma approximately 8 nevi are excised^15^.

Although conventional skin cancer screening based on a total body examination by a trained healthcare professional is not recommended^16^, implementation of an mHealth app might be an intermediate step to consider targeted screening of high risk lesions. In this study use of the app was followed by an increase in claims for (pre)malignancies and could therefore be a potential steppingstone to improve skin cancer detection. However, in its current form, detection by the app includes all cutaneous (pre)malignancies, such as melanomas, keratinocytic carcinomas, and actinic keratosis. These (pre)malignancies have very different morbidities and mortalities^17–20^, and early detection of cutaneous premalignancies such as actinic keratosis is clinically less relevant. A potential negative consequence of population-wide implementation of these type of apps is therefore the risk of overdiagnosis and thus suboptimal use of scarce healthcare resources^21^.

Another issue of using mHealth apps in the general population are the false positive cases. This could lead to anxiety among the users and inappropriate healthcare consumption. In this study, the introduction of the app has led to a significant increase in claims for benign skin tumors and nevi, which can most likely be explained by the suboptimal specificity of 70–78%^9,12,13^. Further increasing the specificity of the investigated app while maintaining a high sensitivity needs to be strived for, to reduce the false positive rate. Nevertheless, even a specificity of 90% will result in a relatively low positive predictive value (PPV) given the low prevalence of skin cancer in a population-based setting^22,23^. A higher PPV is expected in a high risk setting such as patients with a positive (family) history of skin cancer, in certain high prevalent geographic regions such as Australia and USA, or in transplant recipients. Therefore, even though the accuracy will improve as AI-based technology advances, a targeted implementation in high risk groups could be a more beneficial approach^24^.

This study also provides an insight into the healthcare costs related to national implementation of an AI-based mHealth app. The short-term cost-effectiveness analysis estimated the costs to be €2567 per additional skin (pre)malignancy detected by the app, of which €1843 was attributed to claims for benign lesions. Previous research on costs per additional detected skin (pre)malignancy shows a wide range on the cost benefit ratio, depending on the type of intervention^25^. In an Australian study, the cost per additional skin cancer detected by deployment of skin awareness campaigns were 6089 A$ (≈€4141)^26^. While a study from the USA estimated the cost to detect an additional skin (pre)malignancy through total body examination by a dermatologist to be around 2346 US$ (≈€2323)^27^. The advantage of AI-based technology over other interventions is its scalability and that its accuracy will improve as the number of users increases^11^. Over time this can lead to a reduction in the costs as is demonstrated in the ICERS for varying sensitivity and specificity.

In other fields of medicine, costs for screening of different types of cancer can vary wildly, depending on the type of screening, severity of the disease, and the potential benefit. Several countries have national cancer screening programs, for example for breast cancer, colorectal cancer, and cervical cancer^28–31^. The costs per quality of life year (QALY) gained for these types of screening have been described to be as high as 50,000 US$^32–34^. While morbidity and mortality of skin cancer differs substantially between melanoma and keratinocytic cancers, it is lower compared to many other cancers resulting in a higher cost per QALY gained up to 80.000 US$^23,25,35,36^. The difference in healthcare burden and the lack of costs per QALY gained in this study, makes a comparison with other national cancer screening programs difficult. For a more thorough investigation of the cost-effectiveness of AI-based skin cancer detection, a formal cost-effectiveness study is warranted with a longer follow-up and stratification across melanoma and keratinocytic cancers.

This observational study has several limitations. First, the number of skin cancers detected were based on claims data, most likely resulting in an underestimation of the impact of the mHealth app^37^. No data were available on the number, type and stage of skin cancers related to the claims, neither were detailed skin cancer care data available from primary care. Furthermore, despite matching, people using the app may differ from non-users, resulting in residual confounding^38^. People who used the app will likely also include a group that is generally more worried about their skin, with the risk of inducing overdiagnosis. This is also reflected in the fact that mHealth-users who solely received low risk assessments, still more frequently visited a dermatologist for benign skin tumors and nevi. Additionally, the mHealth-users are a relatively younger population and therefore a large part of the elder skin cancer population are not yet part of this intended-use population. This might diminish the potential impact of the app on detection of cutaneous malignancies. Finally, in this study we performed a cross-sectional short-term cost-effectiveness analysis restricted to dermatological healthcare costs. The lack of longitudinal data makes it impossible to estimate the health and cost related impact of early diagnosis of skin cancer. Despite the limitations, this study provides an insight into the impact and costs of implementation of AI-based mHealth apps for skin cancer detection that can contribute to efficient future implementation^39^. Nevertheless, to study the effect of the mHealth app on skin cancer care in more detail and without the aforementioned methodological limitations, a randomized controlled trial is ongoing^40^.

In conclusion, this early evaluation of mHealth-users in a population-based setting demonstrated that introduction of an mHealth app for skin cancer detection was followed by an increased number of claims for (pre)malignancies compared to a group of matched controls. Additionally, mHealth-users also presented with a higher rate of claims for benign lesions. Further improvement in the diagnostic accuracy of the algorithm and a more targeted approach in a high risk population or for high risk lesions, seem to be important steps forward for a successful and cost-effective implementation of mHealth apps for skin cancer diagnostics.

## Methods

### Study design and participants

A retrospective cross-sectional study was conducted to investigate the impact of an mHealth app (SkinVision) on dermatological health care consumption in a large Dutch population. In 2019, a total of 2,213,212 clients of a large insurance company (CZ Groep, Tilburg, The Netherlands) were invited to use this app, free of charge, to evaluate suspicious skin lesions at home^22^. All insurees older than 18 years who completed one or more app based risk assessments of a suspicious skin lesion were included in this study. Users of the app were matched to controls who did not use the app on a 1:3 ratio. Matching criteria were age (categorical 10-year intervals), sex, socio-economic status (SES), residential area, medical history of skin cancer up to four years prior to start of the study, and co-morbidities (continuous number). Most of the mHealth-users could be matched identically to controls (88.3%) on all matching criteria; the remainder of the mHealth-users were matched on all criteria except for residential area. A small portion of mHealth-users (1.1%) that had missing data for their SES were matched to controls for which this data were also missing.

The primary objective of this study was to evaluate the difference in frequency and type of dermatological claims between mHealth-users and controls. Secondary objectives were: 1) to determine differences in therapeutic and diagnostic interventions for suspicious skin lesions, 2) to determine differences in direct healthcare costs, and 3) conduct a short-term cross-sectional cost-effectiveness analysis.

The Medical Ethics Committee of the Erasmus University Medical Center exempted this study from ethical approval (MEC-2020-0385) because it did not fall under the scope of the Medical Research Involving Human Subjects Act (WMO). Data on healthcare claims from the insurer and usage of the app were pseudonymized and linked via a trusted third party (ZorgTTP) to ensure adherence to European privacy guidelines.

### mHealth app

The examined mHealth app (SkinVision, Amsterdam, the Netherlands) is available for Android and iOS smartphones and registered as a CE class I-marked medical device^41^. The app utilizes a convolutional neural network (CNN) that classifies photos of skin lesions as low or high risk of skin cancer and directly provides this information as feedback to the user. The app has an in-build quality check (sharpness, lesions distinguishability, centering) to ensure a photo is suitable for assessment by the CNN. The CNN was recently validated, showing a sensitivity of 87–95% and a specificity of 70–78%^12,13^. To verify the CNN’s assessment, a team of trained teledermatologists evaluated all pictures, and if necessary, adjusted the assessment by an additional message to the user within 48 h. Messages that could be send were either to urgently visit a doctor, visit a doctor within 4 weeks, not to worry and monitor the skin lesion, or take another picture. Use of the app was monitored during a 12-month follow-up period, including data on the CNN’s assessments and teledermatologists’ ratings. For this study, the risk assessments were classified as high risk when either the CNN or teledermatologist assigned high risk, or as low risk when neither the CNN nor the teledermatologist assigned high risk.

### Healthcare claims

Data on dermatological healthcare claims registered during 2019 were collected from the insurance company for all participants. This included all dermatologist-based claims from secondary and tertiary care. One dermatologist-based claim represents for example an intervention performed by a dermatologist (excision), a consultation, chemotherapy, or admittance to a ward. Each claim was registered for a certain diagnosis group (e.g. cutaneous malignancy, or nevi) as defined by the Dutch healthcare authority (NZA)^42^. Claims from primary care were limited to teledermatology consultations, biopsies, and excisions. One GP-based claim for a biopsy or excision was equivalent to one primary care based intervention. GP visits were not included since they were not separately registered as a code. We defined having a positive medical history of skin cancer or cutaneous premalignancies (e.g. actinic keratosis, Bowen’s disease) as a binary variable based on the presence of one or more healthcare claims for (pre)malignancies between 2015 and 2019. The number of unique medications on ATC 5^th^ level was used as a proxy for the number of co-morbidities in accordance with national guidelines^43^. Socio-economic status was based on national data from the Dutch Centraal Planbureau (CPB)^44^, where residential area was determined using anonymized GP codes as a proxy. Costs in the year 2019 related to all included healthcare claims made in a secondary or tertiary dermatology clinic were calculated based on data from the publicly available Dutch national healthcare cost registry (Open DIS data)^42^.

### Statistical analysis

Participants characteristics were described using descriptive statistics. For our main analyses, we tested whether the groups differed in dermatological care consumption, stratified in claims for premalignant skin lesions, malignant skin lesions, benign skin tumors, and nevi. We calculated odds ratios (OR), to determine the likelihood of mHealth-users having either of these dermatological claims compared to controls. Additionally corresponding *p*-values were calculated using Fisher’s Exact Test for Count Data. To test the internal validity of the study design, we compared dermatological claims unrelated to usage of the app, such as varicose veins, since no changes were expected in these claims. To further explore the effect of the mHealth app on dermatological claims we performed a number of follow-up and sensitivity analyses. First, we tested whether the groups differed in the proportion of dermatological claims and claims in a primary care setting by using unpaired two-sided z-tests for independent proportions. Second, to assess if differences in the number of claims were due to the assessments by the app, subgroup analyses were performed for mHealth-users with solely low risk assessments, as well as all mHealth-users with at least one high risk assessment. Third, we tested whether there was a difference in healthcare consumption in a high risk group of mHealth-users and matched controls with a positive medical history for premalignant or malignant skin lesions. Finally, we tested whether the groups differed in their mean healthcare costs using unpaired two-sided *t*-tests. To correct the false discovery rate for multiple testing, *p*-values were adjusted using the Benjamini–Hochberg procedure^45^. Adjusted *p*-values smaller than 0.05 were considered statistically significant. Statistical analyses were performed using the R statistical software (version 4.1.3).

### Short-term cost-effectiveness analysis

A short-term cost-effectiveness analysis was performed to cross-sectionally determine the dermatological costs per additional identified (pre)malignancy by calculating the incremental cost effectiveness ratio (ICER) as given by Eq. (1):

1 $${ICER}=\frac{{Cost}\;{mHealth}-{Cost}\;{standard}\;{of}\;{care}}{{effectiveness}\;{mHealth}-{effectiveness}\;{standard}\;{of}\;{care}}$$

The time horizon was limited to 12 months after receiving free access to the app. The number of unique patients with claims for premalignancies or malignancies were used as a proxy for the number of detected (pre)malignancies. Dermatological costs included all diagnostic and treatment related costs, which were calculated by multiplying the resource use from claims data by the average cost per claim in that year based on the Open DIS data^42^. To account for differences in healthcare cost due to benign skin tumors, nevi, and (pre)malignant skin lesions we calculated the ICER with different components. First, we included the costs for the mHealth app, premalignant, malignant, and benign skin lesions Eq. (2):

2 $${ICER}=\,\frac{({cost}\,m{\rm{Health}}+\,{cost}\,{benign}+{cost}\,({pre}){malignan}{\rm{cies}})\,-({cost}\,{benign}+{cost}\,({pre}){malignan}{cies})}{{detected}\,\left({\rm{pre}}\right){\rm{malignancies}}\; {\rm{mHealth}}-{\rm{{detected}}}\,\left({\rm{pre}}\right){\rm{malignancies}}\,{\rm{of}}\; {\rm{care}}}$$

Second, we included the costs of the mHealth app and only the costs related to benign skin tumors and nevi introduced into the healthcare system Eq. (3):

3 $${ICER}=\,\frac{({cost}\,m{\rm{Health}}+\,{cost}\,{benign}\,{lesions})\,-({cost}\,{benign}\,{lesions})}{{detected}\left({\rm{pre}}\right){\rm{malignancies}}\; {\rm{mHealth}}-{detected}\left({\rm{pre}}\right){\rm{malignancies}}\,{standard}\,{of\; care}}$$

Based on prior research on the accuracy of the app (sensitivity ≈87% and specificity ≈70%) we simulated how the ICER would change with different combinations of sensitivity and specificity^13^.

### Reporting summary

Further information on research design is available in the Nature Research Reporting Summary linked to this article.

## Supplementary information

Supplementary Material

Reporting Summary

## Data Availability

The data collected for this study were combined across multiple healthcare systems through mutual Data Transfer Agreement and under approval of an Institutional Review Board. Therefore, data will not be made publicly available. Study protocol, statistical analysis plan and analytic code can be shared on request for academic purposes. Proposals should be directed to m.wakkee@erasmusmc.nl. To gain access to the study protocol, statistical analysis plan and analytic code, data requestors will need to sign a data access agreement.

## Code availability

The code for the statistical analysis can be shared on request for academic purposes. Proposals should be directed to m.wakkee@erasmusmc.nl.
