# Supplementary material for: An artificial intelligence based app for skin cancer detection evaluated in a population based setting
Source: NPJ Digit Med. 2023 May 20;6:90. doi: 10.1038/s41746-023-00831-w (PMC10199884; doi:10.1038/s41746-023-00831-w)
Supplement: Supplementary file 1 — Supplementary Material [file 41746_2023_831_MOESM1_ESM.pdf]

Supplementary material

Supplemental table 1. Proportion of dermatological healthcare claims for users of the mHealth app that have a positive medical history of skin cancer or premalignant skin lesions (n = 885) and their matched controls (n = 2,655).

	Matched controls (n = 2,655)	mHealth-users (n = 885)	p-value
**Premalignant and malignant skin lesions**			
Premalignant skin lesions			
Percentage, % (n)	25.46 (676)	22.71 (201)	0.138
OR (95% CI)	Ref	0.86 (0.71 - 1.03)	0.138
Malignant skin lesions			
Percentage, % (n)	56.12 (1490)	51.41 (455)	0.041
OR (95% CI)	Ref	0.83 (0.71 - 0.97)	0.041
**Nevi and Benign skin tumors**			
Nevi			
Percentage, % (n)	4.56 (121)	4.86 (43)	0.782
OR (95% CI)	Ref	1.07 (0.73 - 1.54)	0.782
Benign skin tumors			
Percentage, % (n)	2.15 (57)	3.39 (30)	0.087
OR (95% CI)	Ref	1.60 (0.99 - 2.55)	0.087
Unrelated dermatological claims			
Percentage, % (n)	13.71 (364)	10.51 (93)	0.041
OR (95% CI)	Ref	0.73 (0.57 - 0.94)	0.041

Percentages are number of people with a claim per subcategory of claims. P-values are the difference in proportion of claims, calculated using a two proportions z-test or corresponding odds ratio's using Fisher's Exact Test for Count Data. Abbreviations: CI; confidence interval, OR; Odds Ratio, Ref; Reference.

Supplemental Table 2. Total amount of health care related costs per person per diagnosis group for users of the mHealth app (n = 18,960) and a matched cohort (n = 56,880) in the year 2019. Mean costs and 95% confidence interval are reported, the median for all groups was zero.

	Matched controls, mean (95%CI) (€) (n = 56,880)	mHealth-users, mean (95%CI) (€) (n = 18,960)	Difference in € (95%CI)	p-value
Premalignant skin lesions	4.56 (4.09 - 5.04)	6.01 (5.12 - 6.90)	1.45 (0.44 - 2.46)	0.007
Malignant skin lesions	16.32 (15.13 - 17.51)	25.01 (22.52 - 27.49)	8.69 (5.93 - 11.44)	< 0.001
Benign skin tumors	1.03 (0.87 - 1.20)	2.88 (2.44 - 3.33)	1.85 (1.38 - 2.32)	< 0.001
Nevi	2.71 (2.44 - 2.98)	11.05 (10.08 - 12.03)	8.34 (7.34 - 9.35)	< 0.001
Unrelated dermatological claims	18.47 (17.44 - 19.49)	20.01 (17.89 - 22.14)	1.54 (-0.81 - 3.91)	0.211
Total	43.09 (41.40 - 44.79)	64.97 (61.38 - 68.56)	21.88 (17.90 - 25.85)	< 0.001

Costs are mean costs per person for each subcategory based on the number and types of claims multiplied with costs derived from the open DIS data, presented separately for mHealth-users and matched controls. Median for all groups was zero due to the low number of patients with a claim. P-values are based on the difference in mean costs, calculated using an unpaired two-sided t-test. Abbreviations: CI; confidence interval.

Supplemental Figure 1. Boxplot of the distribution of costs per type of claim for a subgroup of mHealth-users who had at least one dermatological claim and controls who had at least one dermatological claim in the year 2019.

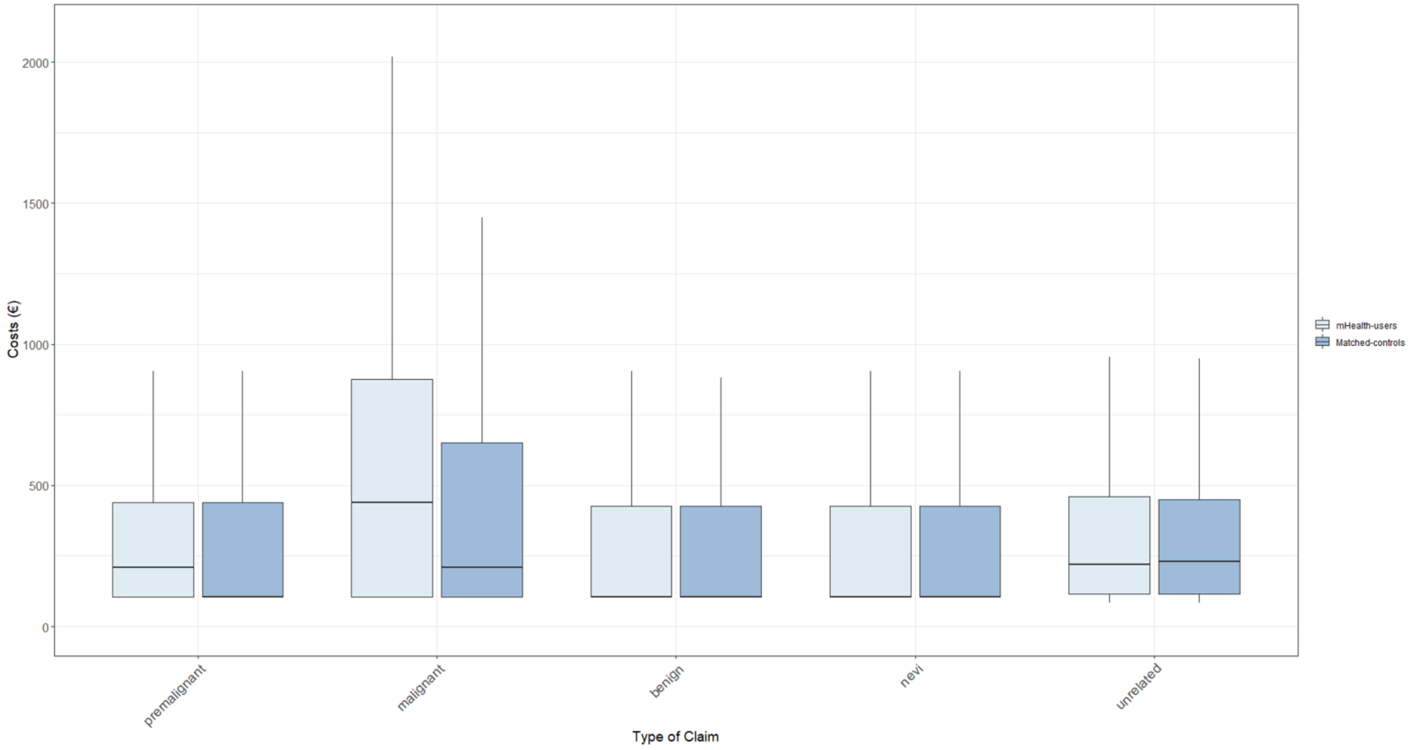

Boxes represent the median costs and interquartile range.

Supplemental Table 3. Total amount of healthcare related costs per person for a subgroup of mHealth-users who had at least one dermatological claim and controls who had at least one dermatological claim in the year 2019.

	Matched controls	mHealth-users	p-value
Premalignant skin lesions			
Number of persons (N)	896	391	
Mean € (95% CI)	289.68 (265.84 - 313.52)	291.48 (259.22 - 323.75)	0.928
Malignant skin lesions			
Number of persons (N)	1785	773	
Mean € (95% CI)	520.05 (490.53 - 549.58)	613.36 (569.39 - 657.33)	<0.001
Benign skin tumors			
Number of persons (N)	279	267	
Mean € (95% CI)	210.88 (188.74 - 233.02)	204.79 (184.77 - 224.82)	0.688
Nevi			
Number of persons (N)	666	850	
Mean € (95% CI)	231.38 (216.04 - 246.72)	246.58 (232.18 - 260.97)	0.156
Unrelated dermatological claims			
Number of persons (N)	2798	1001	
Mean € (95% CI)	375.38 (359.43 - 391.33)	379.05 (345.86 - 412.24)	0.845
Total			
Number of persons (N)	6105	3081	
Mean € (95% CI)	401.49 (401.49 - 414.10)	399.80 (381.85 - 417.75)	0.880

Costs are mean costs per person for each subcategory based on the number and types of claims multiplied with costs derived from the open DIS data, presented separately for mHealth-users and matched controls. P-values are based on the difference in mean costs, calculated using an unpaired two-sided t-test. Abbreviations: CI; confidence interval.
