# Supplementary material for: An artificial intelligence based app for skin cancer detection evaluated in a population based setting
Source: NPJ Digit Med. 2023 May 20;6:90. doi: 10.1038/s41746-023-00831-w (PMC10199884; doi:10.1038/s41746-023-00831-w)
Supplement: Supplementary file 2 — Reporting Summary [file 41746_2023_831_MOESM2_ESM.pdf]

nature portfolio

Corresponding author(s): Marlies Wakkee
Last updated by author(s): 23-02-2023

Reporting Summary

Nature Portfolio wishes to improve the reproducibility of the work that we publish. This form provides structure for consistency and transparency in reporting. For further information on Nature Portfolio policies, see our Editorial Policies and the Editorial Policy Checklist.

Statistics

For all statistical analyses, confirm that the following items are present in the figure legend, table legend, main text, or Methods section.

n/a	Confirmed	
☐	☒	The exact sample size (*n*) for each experimental group/condition, given as a discrete number and unit of measurement
☒	☐	A statement on whether measurements were taken from distinct samples or whether the same sample was measured repeatedly
☐	☒	The statistical test(s) used AND whether they are one- or two-sided<br>*Only common tests should be described solely by name; describe more complex techniques in the Methods section.*
☐	☒	A description of all covariates tested
☐	☒	A description of any assumptions or corrections, such as tests of normality and adjustment for multiple comparisons
☐	☒	A full description of the statistical parameters including central tendency (e.g. means) or other basic estimates (e.g. regression coefficient) AND variation (e.g. standard deviation) or associated estimates of uncertainty (e.g. confidence intervals)
☐	☒	For null hypothesis testing, the test statistic (e.g. *F*, *t*, *r*) with confidence intervals, effect sizes, degrees of freedom and *P* value noted<br>*Give P values as exact values whenever suitable.*
☒	☐	For Bayesian analysis, information on the choice of priors and Markov chain Monte Carlo settings
☒	☐	For hierarchical and complex designs, identification of the appropriate level for tests and full reporting of outcomes
☐	☒	Estimates of effect sizes (e.g. Cohen's *d*, Pearson's *r*), indicating how they were calculated

Our web collection on statistics for biologists contains articles on many of the points above.

Software and code

Policy information about availability of computer code

Data collection	The data in this study were collected from SkinVision (Amsterdam, the Netherlands) and CZ Groep (Tilburg, The Netherlands) and matched via a trusted third party (ZorgTTP). Data was processed using R statistical software (version 4.1.3). Healthcare cost were obtained from the Dutch national healthcare cost registry (Open DIS data): https://www.opendisdata.nl
Data analysis	R statistical software (version 4.1.3)

For manuscripts utilizing custom algorithms or software that are central to the research but not yet described in published literature, software must be made available to editors and reviewers. We strongly encourage code deposition in a community repository (e.g. GitHub). See the Nature Portfolio guidelines for submitting code & software for further information.

Data

Policy information about availability of data

All manuscripts must include a data availability statement. This statement should provide the following information, where applicable:

- Accession codes, unique identifiers, or web links for publicly available datasets
- A description of any restrictions on data availability
- For clinical datasets or third party data, please ensure that the statement adheres to our policy

The data collected for this study were combined across multiple healthcare systems through mutual Data Transfer Agreement and under approval of an Institutional Review Board. Therefore, data will not be made publicly available. Study protocol, statistical analysis plan and analytic code can be shared on request for academic

purposes. Proposals should be directed to m.wakkee@erasmusmc.nl. To gain access to the study protocol, statistical analysis plan and analytic code, data requestors will need to sign a data access agreement.

Human research participants

Policy information about studies involving human research participants and Sex and Gender in Research.

Reporting on sex and gender	Data on sex were obtained from the health insurer. It was used as one of the criteria to match mHealth-users and controls to ensure a similar distribution of sex in both groups. Descriptive statistics were used to report the distribution of sex in the study population. Data on gender was not available and therefore not reported.
Population characteristics	18,960 mHealth-users were matched to 56,880 controls. Users of the app and their controls had a mean age of 48.4 years (SD 14.0), 53.2% were female, and 42.6% were classified as middle SES. The majority did not have a history of skin cancer (95.3%) and were healthy individuals (median number of comorbidities 0 (IQR 0-0))
Recruitment	In this retrospective study, we included all insurees of a large insurance company (CZ Groep, Tilburg, The Netherlands) older than 18 years who completed one or more app based risk assessments of a suspicious skin lesion in the year 2019. Users of the app were matched to controls who did not use the app on a 1:3 ratio.
Ethics oversight	The Medical Ethics Committee of the Erasmus University Medical Center exempted this study from ethical approval (MEC-2020-0385) because it did not fall under the scope of the Medical Research Involving Human Subjects Act (WMO).

Note that full information on the approval of the study protocol must also be provided in the manuscript.

Field-specific reporting

Please select the one below that is the best fit for your research. If you are not sure, read the appropriate sections before making your selection.

☐ Life sciences ☒ Behavioural & social sciences ☐ Ecological, evolutionary & environmental sciences

For a reference copy of the document with all sections, see nature.com/documents/nr-reporting-summary-flat.pdf

Behavioural & social sciences study design

All studies must disclose on these points even when the disclosure is negative.

Study description	A retrospective cross-sectional study was conducted to quantitatively investigate the impact of an mHealth app (SkinVision) on dermatological health care consumption in a large Dutch population
Research sample	In 2019, a total of 2,213,212 clients of a large insurance company (CZ Groep, Tilburg, The Netherlands) were invited to use this app, free of charge, to evaluate suspicious skin lesions at home. All insurees older than 18 years who completed one or more app based risk assessments of a suspicious skin lesion were included in this study. Users of the app were matched to controls who did not use the app on a 1:3 ratio.
Sampling strategy	This was a real-word retrospective study. The study sample was based on all insurees from a large health insurer who downloaded and succesfully used an mHealth app in 2019. To compare results to a control group, mHealth-users were matched on a ratio of 1:3 to insurees who did not use the app.
Data collection	Data on usage of the app was collected over a 12-month follow-up period, including data on the CNN's assessments and teledermatologists' ratings. Data on dermatological healthcare claims registered during 2019 were collected from the insurance company for all participants. Costs in the year 2019 related to all included healthcare claims made in a secondary or tertiary dermatology clinic were calculated based on data from the publicly available Dutch national healthcare cost registry (Open DIS data).
Timing	Data was retrospectively collected to evaluate the difference in dermatological healthcare consumption in 2019.
Data exclusions	We excluded 1817 participants who changed health insurer, because the impact on care could not be evaluated. Additionally we excluded participants who downloaded the app but did not have any (succesfull) ratings.
Non-participation	Not applicable
Randomization	Participants were not allocated into experimental groups

Reporting for specific materials, systems and methods

We require information from authors about some types of materials, experimental systems and methods used in many studies. Here, indicate whether each material, system or method listed is relevant to your study. If you are not sure if a list item applies to your research, read the appropriate section before selecting a response.

Materials & experimental systems

n/a	Involved in the study
☒	☐ Antibodies
☒	☐ Eukaryotic cell lines
☒	☐ Palaeontology and archaeology
☒	☐ Animals and other organisms
☒	☐ Clinical data
☒	☐ Dual use research of concern

Methods

n/a	Involved in the study
☒	☐ ChIP-seq
☒	☐ Flow cytometry
☒	☐ MRI-based neuroimaging
